# Formulation, Characterization, and Lipolysis Properties of Lycopene-Loaded Self-Emulsifying Delivery Systems Based on Different Lipids

**DOI:** 10.3390/foods14234162

**Published:** 2025-12-04

**Authors:** Siao-Jhen Lin, Yi-Chan Chiang, Kai-Min Yang, Po-Yuan Chiang

**Affiliations:** 1 Department of Food Science and Biotechnology, National Chung Hsing University, 145 Xingda Road, South District, Taichung City 40227, Taiwan; 2 Department of Food Science, National Quemoy University, No. 1, University Road, Jinning Township, Kinmen County 89250, Taiwan

**Keywords:** self-emulsifying delivery system, lycopene, nonionic surfactants, lipolysis, bioaccessibility

## Abstract

Lycopene is a naturally potent lipophilic antioxidant, which limits its bioavailability for absorption during intestinal digestion. Therefore, this study utilized a self-emulsifying delivery system (SEDS) to enhance the solubility and bioavailability of lycopene and investigated the effects of nonionic surfactant mixtures at varying hydrophilic–lipophilic balance (HLB) values and surfactant-to-oil ratios (SORs) on SEDS using oleic acid (OA), medium-chain triglycerides (MCTs), and sunflower oil (SO) as oil matrices. The resulting water-in-oil-in-water emulsions exhibited droplet sizes (181.70 to 572.27 nm), polydispersity indices (0.29 to 0.86), and ζ-potentials (−22.90 to −53.70 mV), with stability varying according to the type of oil and formulation parameters. Antioxidant activities of SO-based SEDS were higher compared to MCT-based and OA-based ones due to lycopene loading increase. *In vitro* simulated intestinal digestion revealed differences in lipolysis kinetics, with MCT-based lycopene-loaded SEDS exhibiting enhanced cumulative release and bioaccessibility in the duodenal (1.1–2.1 mEq/g) and jejunal (1.6–2.2 mEq/g) segments. This study revealed a comprehensive strategy encompassing lycopene extracts, SEDS preparation, quality indices, lipolysis dynamics, and proximal intestine solubilization amounts that successfully enhanced lycopene bioavailability. Optimized MCT-based lycopene-loaded SEDS with high HLB (10.72) and SOR (1.00) enhanced hydrophobic bioactive delivery efficiency, offering a novel low-energy strategy for developing functional supplements.

## 1. Introduction

In recent years, the global nutraceutical market has experienced sustained growth driven by increasing consumer health awareness, which has heightened demand for delivery systems of natural active pharmaceutical ingredients [1]. Lycopene is a lipophilic carotenoid widely present in red, pink, and orange fruits and vegetables, such as tomatoes, watermelons, and papayas [2,3]. Its molecular structure contains 2 non-conjugated double bonds and 11 conjugated bonds, conferring a highly conjugated unsaturated structure that enables effective neutralization of reactive oxygen species and singlet oxygen, thereby scavenging free radicals and exhibiting potent antioxidant activity [4]. Lycopene’s ability to quench singlet oxygen surpasses that of β-carotene and vitamin E, emphasizing its role in maintaining redox balance [5]. It has also garnered attention for its potential in ameliorating chronic conditions such as prostate cancer, ocular diseases, cardiovascular diseases, and neurodegenerative disorders [5,6]. However, all-*trans* lycopene is a long chain bioactive, resulting in lower solubility during intestinal digestion and thereby limiting the formation of mixed micelles. Lycopene is also highly sensitive to light, heat, and oxygen, posing challenges to its use in food applications due to low water solubility, instability, thermal sensitivity, and limited bioavailability [7,8]. Previous studies have demonstrated that emulsification can improve the bioavailability of lycopene [5,9].

As a low-energy emulsification technique, self-emulsification promotes spontaneous curvature of surfactants, enabling the spontaneous formation of oil-in-water (O/W) or water-in-oil-in-water (W/O/W) emulsion systems under mild mechanical stirring, offering high stability and environmental friendliness [10,11]. Differing from traditional emulsification methods, self-emulsifying delivery systems (SEDS) comprise oils, nonionic surfactants, or cosolvents. During *in vitro* simulated intestinal digestion, due to the surfactants’ excellent amphiphilic properties and ability to reduce interfacial tension, they rapidly and spontaneously form nano-scale emulsions in the aqueous phase [12,13]. Due to their high biocompatibility and stability in digestive fluids, nonionic surfactants are commonly used in food- and drug-grade SEDS to enhance their spontaneous self-emulsification efficiency [14,15,16]. SEDS can achieve spontaneous emulsification through gastric and intestinal peristalsis, not only improving the loading and absorption rates of hydrophobic APIs but also enhancing thermal stability and retention rates, making them a key carrier in functional foods and nutraceuticals in recent years [17]. Various SEDS formulations utilize the hydrophilic–lipophilic balance (HLB) of surfactants and the surfactant-to-oil ratio (SOR) [18].

SEDS have been successfully employed to deliver various hydrophobic phytochemicals, including curcumin [19], lycopene [20], and cannabidiol [21]. However, gaps remain in the ability of related *in vitro* assays to assess lipolysis dynamics during *in vitro* intestinal fluid digestion. Therefore, this study develops lycopene-loaded SEDS (LL-SEDS) using varying ratios of nonionic surfactants and utilizes a continuous simulated intestinal lipolysis model to investigate the release dynamics of free fatty acids (FFAs) and the solubilization amount (SA) in the proximal intestine, further exploring its delivery characteristics for enhancing bioavailability. This study also evaluates the lycopene loading capacity and molecular interactions, as well as assesses the spontaneous emulsification characteristics, median droplet size (D_50_), and polydispersity index (PDI). The findings may provide guidance for future low-energy preparation of delivery carriers for hydrophobic compounds.

## 2. Materials and Methods

### 2.1. Materials and Chemicals

Five percent lycopene extract (LE) powder was obtained from Xin Kang Le Co., Ltd., Taichung City, Taiwan. Oleic acid (OA) was obtained from Shen Chiu Enterprise (Taichung City, Taiwan). Sunflower oil (SO) was obtained from Standard Foods Corporation (Taichung City, Taiwan). Medium-chain triglycerides (MCTs), polysorbate 80 (Tween 80 [T80]), and sorbitan monooleate (Span 80 [S80]) were obtained from Yu Ba Enterprise (Taipei City, Taiwan). OA, SO, and MCTs were edible-grade oils. All chemicals were analytical grade and obtained from Sigma-Aldrich (St. Louis, MO, USA). All solutions were prepared using deionized water with a resistivity of at least 18.2 MΩ·cm^−1^.

### 2.2. Quality Indices of SEDS

#### 2.2.1. Miscibility Test

Emulsifiers T80 and S80 were mixed at ratios of 4:1, 3:2, 1:1, 2:3, and 1:4 (*w*/*w*). Oils MCTs, OA, and SO were combined with the mixed emulsifiers at ratios of 2:1, 3:2, and 1:1 (*w*/*w*) under magnetic stirring (300 rpm) at room temperature for 24 h to prepare SEDS formulations with different HLB values (Equation (1)) and SORs (Equation (2)). Formulations forming a homogeneous phase without separation were selected for evaluation of self-emulsification. Pseudo-ternary phase diagrams were constructed using CHEMIX software (version 12.5; Codelite Ltd., London, UK), with black dots indicating miscible points. Then, each miscible preconcentrate was diluted at a 1:1000 (*w*/*w*) ratio in 5 mM phosphate buffer solution (PBS, pH 7.4) at 25 °C and stirred magnetically (300 rpm) at room temperature to assess self-emulsification ability. Preparations that spontaneously dispersed into rapid, uniform emulsions were classified as good or moderate and marked with orange dots in the pseudo-ternary phase diagram [22].

(1) $${HLB}_{mix}=\sum_{i=1}^{n}\left(\frac{W_{i}}{W_{total}}\times {HLB}_{i}\right)$$

(2) $$SOR=\frac{W_{surfactant}}{W_{oil}}$$

*HLB*_mix_ = The final HLB value of the mixed surfactants.
*HLB*_i_ = The individual HLB values of the surfactants.
*W*_i_ = The respective amounts of surfactants (expressed as a weight or molar ratio).
*SOR* = The surfactant-to-oil ratio.
*W*_surfactant_ = The weight (or volume) of the surfactant.
*W*_oil_ = The weight (or volume) of the oil.

#### 2.2.2. Solubility of Lycopene

First, 2 g of SO, MCTs, S80, T80, or SEDS was weighed into 10 mL beakers, followed by 0.5 g of LE, and then magnetically stirred (300 rpm) at ambient temperature for 24 h. Then, the mixture was centrifuged (5000 rpm, 5 min), and 0.2 g of the supernatant was diluted with 2 mL of ethyl acetate (EA), vortexed for 10 s, and filtered through a 0.22 μm membrane to ensure no precipitate before high-performance liquid chromatography–ultraviolet (HPLC-UV) analysis. The HPLC-UV system consisted of an autosampler (PN5300; Postnova, Salt Lake City, UT, USA), a chromatographic pump (Chromaster 5110; Hitachi Co., Tokyo, Japan), a Mightysil RP-18GP column (250 mm, 4.6 mm i.d., 5.0 μm; Kanto Co., Tokyo, Japan), and an ultraviolet–visible detector (Chromaster 5420; Hitachi Co., Tokyo, Japan). The mobile phase gradient began with 70% A (75% methanol in deionized water) and 30% B (EA), increasing to 90% A over 11.5 min, decreasing to 30% A over 3.9 min, holding for 3.8 min, and then returning to 70% A over 3.9 min. Samples (15 μL) were injected at 25 °C with a flow rate of 1.00 mL/min. The detection wavelength was set at 450 nm. Lycopene was identified by comparing retention times (min) with external standards and quantified using calibration curves (2000, 500, 250, and 12.5 mg/kg; *R*^2^ > 0.999) [23].

#### 2.2.3. Fourier-Transform Infrared (FTIR) Spectroscopy and Unsaturation Degree (UD)

All SEDS (including LL-SEDS) were analyzed using an FTIR spectrometer equipped with an MCT detector (Nicolet 6700; Thermo Fisher Scientific, Waltham, MA, USA). Spectra were acquired in the wavenumber range of 4000–650 cm^−1^ at a resolution of 2 cm^−1^/30 s. The UD was monitored using Equation (3), a method commonly employed to assess the unsaturation of vegetable oil and alterations in lipid organization [24,25]:

(3) $$UD=A_{3006}/A_{2922}$$

A_3006_ = The absorbance at wavenumber 3006 cm^−1^.
A_2922_ = The absorbance at wavenumber 2922 cm^−1^.

### 2.3. Quality Indices of W/O/W Emulsion

#### 2.3.1. Microstructure

First, 1 g of the SEDS formulation was added dropwise to 10 g of 5 mM PBS (pH 7.4) and vortexed at 600 rpm for 1 min. Then, its stereoscopic optical microstructure was examined under a research surgical microscope (Eclipse E400; Nikon Co., Tokyo, Japan).

#### 2.3.2. Measurement of Droplet Size, PDI, and ζ-Potential

First, 0.1 g of the SEDS formulation was added dropwise to 100 mL of 5 mM PBS and stirred (300 rpm) for 10 min. Then, an appropriate amount of the emulsion was placed in a disposable cuvette and analyzed for micelle characteristics at 25 °C using a laser nanoparticle size and zeta potential analyzer (Zetasizer Nano-ZS; Malvern Panalytical, Malvern, UK), with deionized water as the continuous phase, a viscosity of 0.8872 cP, and a refractive index of 1.330 [25].

#### 2.3.3. Turbidity

First, 0.1 g of the SEDS formulation was added dropwise to 100 mL of 5 mM PBS (pH 7.4) and stirred (300 rpm) for 10 min. Then, 200 μL was placed in a disposable cuvette and its absorbance at 600 nm was measured using a spectrophotometer (U-2800-A; Hitachi Co., Tokyo, Japan). Based on their optical density at 600 nm, emulsions were classified as transparent (<0.05), bluish/semi-transparent (0.05–0.1), turbid (0.1–0.3), or milky (>0.3) [26].

### 2.4. Antioxidant Capacities and Lipolysis Dynamics

#### 2.4.1. 2,2′-Azino-Bis (3-Ethylbenzothiazoline-6-Sulfonic Acid) (ABTS) Radical Cation Scavenging Activity

An ABTS radical cation solution (7 mM ABTS and 2.45 mM potassium persulfate (K_2_S_2_O_8_), 2:1 *v*/*v*) was prepared and stored in the dark for 12–16 h. Then, the solution was diluted with 100 mM Tris-HCl buffer (pH 7.4) to achieve an absorbance at 734 nm (A_734_) of 0.700 ± 0.02. Next, 240 µL of the diluted ABTS solution was mixed with 10 µL of SEDS extracts (1 g of SEDS dissolved in 10 mL of 80% ethanol), and the A_734_ value was determined using a microplate reader (SPECTROstar Nano; BMG Labtech Co., Ortenberg, Germany) after 30 min. Trolox was used as the standard, quantified via a calibration curve to dry weight (mg/g). The scavenging rate was calculated using Equation (4) [27]:

(4) $$ABTS\;free\;radical\;scavenging\;(\%)=(1-A_{sample}/A_{control})\times 100$$

*A*_sample_ = The absorbance of the SEDS solution.
*A*_control_ = The absorbance of the analyte solution alone.

#### 2.4.2. Ferric Reducing Antioxidant Power (FRAP)

FRAP reagent was mixed with 300 mM acetate buffer (pH 3.6), 10 mM 2,4,6-tripyridyl-s-triazine (in 40 mM HCl), and 20 mM iron(III) chloride (in double-distilled water) at a 10:1:1 ratio. Next, 20 μL of SEDS extracts was added to 150 μL of FRAP reagent and incubated at 37 °C in the dark for 10 min. Then, the absorbance was measured at 593 nm using the same microplate reader. Trolox was used as the standard, quantified via calibration curve to dry weight (mg/g) [28].

#### 2.4.3. Lipolysis Dynamics of Intestinal Fluids and Solubilization Amount

Lipolysis was evaluated using an automatic titrator (AUT-701; DKK-TOA Corp., Osaka, Japan) at 37.5 °C (pH 7.0) with 5 mM PBS (pH 7.4). Self-emulsified dispersions (1:1000, *w*/*w*) were equilibrated for 5 min at 150 rpm, followed by the initiation of digestion through the addition of 40 mg of pancreatin lipase (109.3 USP) and bovine bile extract. This assay replicated transit through the small intestine: the duodenum (0–30 min), the jejunum (30–90 min), and the ileum (90–180 min). FFAs (mEq/g) were quantified by measuring the consumption of 0.1 M NaOH and adjusting for the blank value. The solubilization amount was determined using Equation (5), based on the cumulative release amounts of segments from the duodenum and jejunum [29].

(5) $$Solubilization\;amount\;(mEq/g)=R_{duodenum}+R_{jejunum}$$

*R*_duodenum_ = The cumulative release amount of FFAs between 0 and 30 min.
*R*_jejunum_ = The cumulative release amount of FFAs between 30 and 90 min.

### 2.5. Statistical Analysis

All results are expressed as mean ± standard deviation. Data were compared between groups using one-way analysis of variance with post hoc Duncan’s multiple range tests using SPSS (version 19.0; IBM Corp., Armonk, NY, USA), with a *p*-value of <0.05 considered statistically significant. Principal component analysis (PCA) and agglomerative hierarchical clustering were conducted utilizing XLSTAT software (version 2025.1; Addinsoft, NY, USA).

## 3. Results and Discussion

### 3.1. Formulation Screening

SEDS consist of an oil phase, nonionic surfactants, and co-surfactants. Initially, a homogeneous phase must form under anhydrous conditions to confirm miscibility, enabling the formation of stable micron- or nano-scale O/W or W/O/W emulsion droplets upon addition of a substantial aqueous phase [30,31]. This study employed high-HLB T80 (polysorbate 80, HLB = 15.0) and low-HLB S80 (sorbitan monooleate, HLB = 4.3) as surfactants, mixed at ratios of 4:1, 3:2, 1:1, 2:3, and 1:4 (*w*/*w*), yielding mixed HLB values of approximately 12.86, 10.72, 9.65, 8.58, and 6.44, respectively. These were then combined with three oil phases, SO (long-chain triglycerides), MCTs, and OA (fatty acid), at SORs of 0.50, 0.67, or 1.00, corresponding to oil–surfactant ratios of 2:1, 3:2, and 1:1 (*w*/*w*), to prepare homogeneous SEDS.

**Figure 1:**
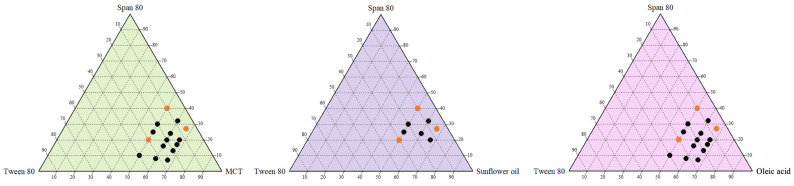
Pseudo-ternary phase diagram of self-emulsifying delivery system. Miscible formulations are denoted by solid black dots, whereas orange dots indicate the groups selected through screening.

In the pseudo-ternary phase diagram (Figure 1), all miscible SEDS formulations are marked by solid black dots, with orange dots indicating secondary screening groups. SO formed single phases less readily at low SORs, resulting in fewer solid black dots in the diagram. Thus, the SOR needed to be increased to 0.67–1.00 and the HLB reduced to 6.44–9.65 to improve compatibility [32,33]. In contrast, MCTs and OA formed single phases across all examined HLB and SOR conditions, attributed to the hydrophobicity and high viscosity of SO, necessitating the use of more surfactants and lower-HLB S80. MCTs and OA function as both oils and surfactant-like components, improving interfacial tension upon aqueous addition and facilitating microemulsion/self-emulsification [30,34,35,36].

### 3.2. Lycopene Solubility in the Oils and Surfactants

**Figure 2:**
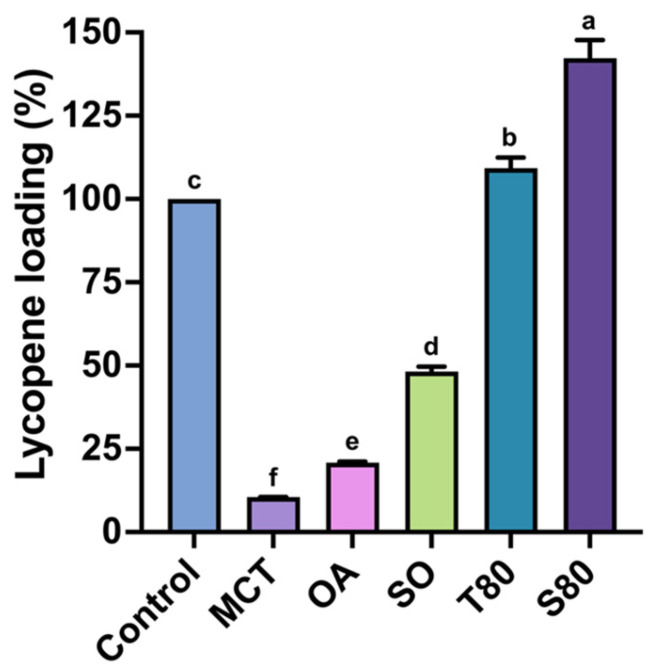
The loading rate of lycopene. Data are presented as means ± standard deviations (*n* = 3). Different letters (a–f) indicate statistically significant differences in the same column (*p* < 0.05). MCT, OA, SO, T80, and S80 denote medium-chain triglycerides, oleic acid, sunflower oil, Tween 80, and Span 80, respectively.

To compare carrier capacities for the maximum dissolution of lycopene in LE, excess LE was added to three oil phases (MCTs, OA, and SO) and two nonionic surfactants (T80 and S80), and lycopene loading was quantified via HPLC-UV analysis (Figure 2). The surfactants exhibited significantly higher maximum loading of lycopene than the edible oils (*p* < 0.05). Using pure EA extraction as a positive control (100%), S80 and T80 achieved lycopene loadings of 142.26% and 109.27%, respectively, indicating that nonionic surfactants provide favorable microenvironments for hydrophobic dissolution, consistent with reports on Tweens or Spans enhancing carotenoid dissolution/delivery and nonionic surfactant-based microemulsions improving lycopene solubility/stability [37,38,39].

Between the surfactants, S80 exhibited higher maximum loading than T80. Given their shared hydrophobic tails (C18:1) but different HLB (S80 = 4.3, T80 = 15.0), this difference may be attributed to S80’s larger hydrophobic region and lower polarity, favoring lycopene retention. Similar HLB-dependent solubility has been observed in Tween–Span mixtures [40].

Among the edible oils, the maximum loading of lycopene was highest with SO (48.13%), followed by OA (20.82%) and MCT (10.49%), indicating that fatty acid composition and polarity critically influence lycopene solubility and loading. Previous studies have reported substantial lipid effects on lycopene solubility, noting that MCTs, linoleic acid, or OA enhance carotenoid extraction efficiency. Our study provides further formulation compositions with SORs and HLB values for subsequent lycopene loading research [41,42].

Overall, selecting nonionic surfactants with appropriate HLB ranges, such as more hydrophobic S80, can stabilize and significantly enhance lycopene dissolution. Oil phase selection in LL-SEDS is also crucial, balancing edibility, non-polarity, and unsaturation [37,43]. Vasconcelos et al. [44] reported that the hydrophobic microstructure formed by SEDS, consisting of nonionic surfactants and an oil phase, can increase the solubility of hydrophobic bioactives. Therefore, the following section further investigates the real and theoretical loading of lycopene.

### 3.3. Formulation and FTIR Measurement of LL-SEDS

The lycopene loadings in SEDS formulations created from different edible oils under three parameters are listed in Table 1. Actual loadings exceeded theoretical loadings in all formulations, with increases ranging from 7.07% to 100.08%, indicating that surfactant synergy enhances lycopene solubility [45]. Among H6S1L formulations composed of different edible oils, both theoretical and actual lycopene loadings were relatively high, primarily due to higher S80 proportions and strong lipophilicity aiding lycopene stabilization. Conversely, SH10S1L exhibited the highest actual loading (*p* < 0.05) at 29.87 ± 0.16 mg/g, followed by MH6S1L at 28.99 ± 0.47 mg/g, with no significant intra-group differences. This observation suggests SO-based SEDS with equal surfactant proportions (HLB = 10.72) or MCT-based SEDS with equal surfactant proportions (HLB = 6.44) as suitable candidates for lycopene loading.

**Figure 3:**
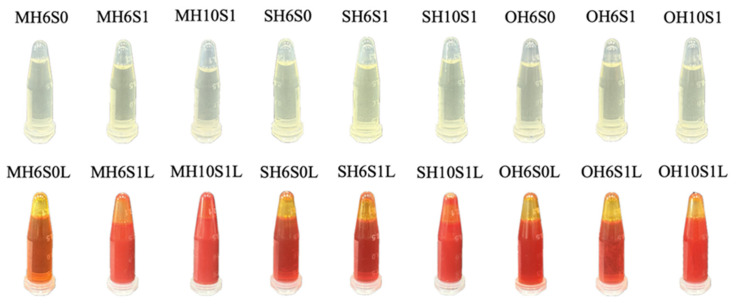
The appearance of lycopene-loaded self-emulsifying delivery system. M, S, O, H6, H10, S0, S1, and L denote medium-chain triglyceride, sunflower oil, oleic acid, HLB = 6.44, HLB = 10.72, SOR = 0.50, SOR = 1.00, and lycopene-loading, respectively.

Differences in appearance in SEDS before and after the addition of lycopene are shown in Figure 3. Unloaded SEDS were mostly transparent, turning varying shades of red-orange upon addition, indicating lycopene dissolution into SEDS. Variations in transparency after the addition of lycopene reflect the influences of HLB and SOR on LL-SEDS stability. H10S1L formulations across the three edible oils exhibited decreased transparency, attributed to the high HLB (more hydrophilic) of the surfactant reducing oil droplet dispersibility, leading to insufficient lycopene compatibility in the oil phase and reduced transparency [46]. The transparency of the MH6S1L formulation also decreased, attributed to the low HLB biasing SEDS toward lipophilicity, which increases edible oil solubility but potentially causes droplet aggregation or broadened size distribution at high SOR. Given lycopene’s hydrophobicity, its dispersion efficiency in MCTs is inherently limited; uneven interfacial arrangement or thick droplet coverage may induce instability, increasing light scattering [47].

**Figure 4:**
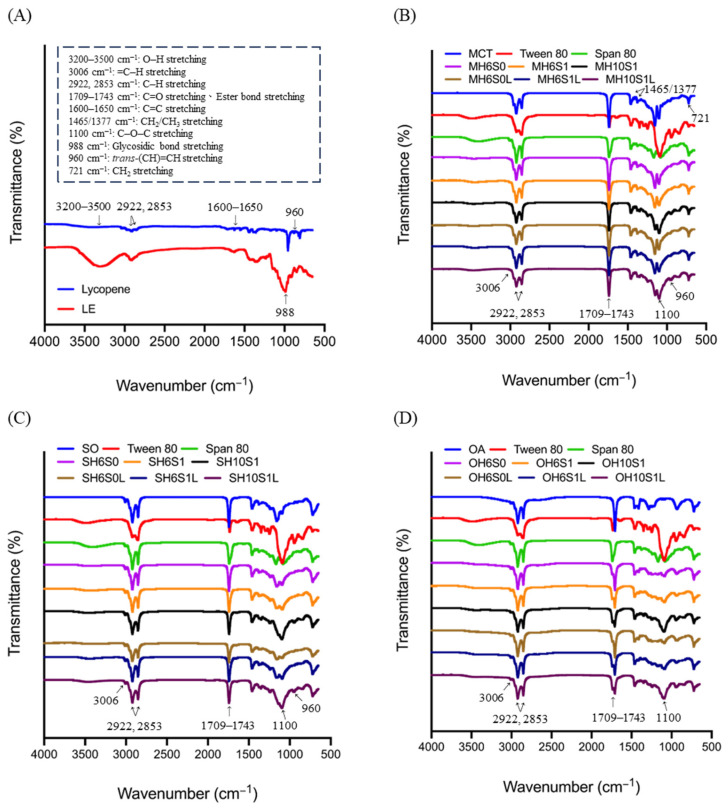
The FTIR spectrum of lycopene-loaded self-emulsifying delivery system. (**A**) Lycopene standard and commercial extracts (**B**) MCT-based LL-SEDS (**C**) SO-based LL-SEDS (**D**) OA-based LL-SEDS M, S, O, H6, H10, S0, S1, and L denote medium-chain triglyceride, sunflower oil, oleic acid, HLB = 6.44, HLB = 10.72, SOR = 0.50, SOR = 1.00, and lycopene-loading, respectively.

FTIR spectroscopy was employed to characterize the functional groups of the components and to examine potential chemical interactions after lycopene loading. The spectra of the lycopene standard and tomato extract (LE) exhibited aliphatic C–H stretching vibrations at 2922 and 2853 cm^−1^ (Figure 4A). The LE spectrum exhibited a broad O–H stretching band at 3200–3500 cm^−1^, attributed to hydrogen-bonded polysaccharides and polyphenols, as well as glycosidic bond vibrations near 988 cm^−1^. The lycopene standard exhibited a distinct all-*trans* =CH out-of-plane bending at approximately 960 cm^−1^, along with weak conjugated C=C stretching signals in the range of 1600–1650 cm^−1^, which may be obscured by matrix effects in complex samples [23,48,49,50].

Consistent lipid characteristics were identified in all SEDS formulations with varying oil phases (Figure 4B–D), including a pronounced ester C=O stretching at 1738–1743 cm^−1^ (attributable to triglycerides and sorbitan/polyoxyethylene sorbitan esters), CH_2_ stretching at 2922/2853 cm^−1^, CH_2_/CH_3_ bending at 1465/1377 cm^−1^, and long-chain CH_2_ rocking at approximately 721 cm^−1^. The band around 1100 cm^−1^, associated with poly (ethylene oxide) C–O–C stretching, was significant in formulations that included T80, while it was weak or absent in formulations without polyoxyethylene groups, such as S80 and OA [51,52].

In MCT-based SEDS (Figure 4B), minimal olefinic =C–H stretching was observed at approximately 3006 cm^−1^, which aligns with the saturated composition of MCTs. Following lycopene loading, intensity increased near 960 cm^−1^, corresponding to the characteristic band of lycopene. The carbonyl and C–O–C regions exhibited minimal changes (peak shifts < 2 cm^−1^), indicating microenvironmental perturbations rather than covalent modifications [53]. SO-based SEDS (Figure 4C) exhibited a more prominent band around 3006 cm^−1^, attributable to the increased unsaturation level associated with linoleic acid richness. The ester C=O at 1739 cm^−1^ and C–O–C at 1100 cm^−1^ (from T80) were predominant, with lycopene loading resulting in only minor modifications or shoulders near 960 cm^−1^ [51,54]. OA-based SEDS (Figure 4D) exhibited overlapping C=O bands, with ester contributions observed at approximately 1739 cm^−1^ from surfactants and carboxylic acid C=O at 1709 cm^−1^ from dimeric OA. The 1100 cm^−1^ band was weak in pure OA, which lacks ether linkages, but was present in formulations containing T80. Lycopene loading caused slight intensity redistributions in the fingerprint region, with no new peaks appearing. The FTIR spectra indicate that lycopene is physically solubilized within the self-emulsifying matrices, showing no evidence of covalent interactions [55].

**Figure 5:**
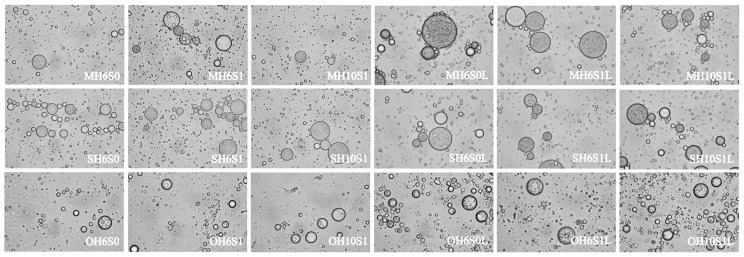
The emulsion droplet microstructure of lycopene-loaded self-emulsifying delivery system. M, S, O, H6, H10, S0, S1, and L denote medium-chain triglyceride, sunflower oil, oleic acid, HLB = 6.44, HLB = 10.72, SOR = 0.50, SOR = 1.00, and lycopene-loading, respectively. All micrographs were acquired at same magnification.

The stereoscopic optical microstructure of various LL-SEDS is shown in Figure 5. Microscopic observation revealed larger D_50_ and uneven distribution in SO-based formulations, whereas the interfacial membranes remained relatively stable with predominantly spherical droplets. This stability may be attributed to the triglyceride composition of SO, which offers stable hydrophobic cores and minimal interfacial interference, thereby supporting droplet integrity. In contrast, OA-based formulations exhibited smaller droplets and thinner interfacial membranes, attributed to OA being a free unsaturated fatty acid, which impedes tight interfacial packing and consequently diminishes structural stability [56]. MCT-based formulations demonstrated intermediate droplet size and interfacial stability. Lycopene SEDS exhibited W/O/W double-emulsion structures, which function as physical barriers to minimize lycopene exposure to external factors such as light and oxygen [57].

### 3.4. Quality Assessment and Structural Characteristics of W/O/W Emulsion

To evaluate formulation effects on the quality of LL-SEDS, the D_50_, PDI, and turbidity (A_600_) were assessed in dispersed PBS (Table 2). Overall, the type of oil significantly influenced the distribution of droplet sizes in the emulsion. MCT-based SEDS had D_50_ mainly between 260.37 and 572.27 nm, while SO-based SEDS generally had D_50_ exceeding 400 nm, attributed to higher-molecular-weight triglycerides reducing emulsification efficiency, with viscosity also being key to configuration formation [58]. OA-based SEDS had the smallest D_50_, approximately 181.70–322.03 nm, possibly due to OA’s fatty acid structure and higher fluidity aiding interfacial tension reduction [59].

PDI measures the range of droplet sizes in emulsions, a key indicator of stability and quality. A PDI below 0.3 indicates a uniform distribution and stable systems with consistent, narrow droplet sizes, ideal for stability. A higher PDI, above 0.7, indicates polydispersity with broad size ranges, often leading to lower stability from aggregation or coalescence [60]. For the MCT-based SEDS, the PDI ranged from 0.33 to 0.86, with MH10S1 more uniform (PDI: 0.33 ± 0.04) and MH6S1 less stable (PDI: 0.86 ± 0.19), indicating SEDS stability depends highly on the SOR and surfactant HLB (Table 2). As the medium-chain saturated fatty acids in MCT give it stronger hydrophobicity than OA, it is more miscible with surfactants containing higher HLB and can spontaneously disperse to form nanoemulsions with low PDI [61]. For the SO-based SEDS, the PDI was more concentrated, mostly between 0.45 and 0.60, possibly due to the higher proportions of polyunsaturated fatty acids, which promote fluidity and hydrophobicity, enabling stable emulsions across SOR with reduced PDI variation [62]. For the OA-based SEDS, the PDI ranged from 0.29 to 0.80, showing unstable dispersion similar to MCT-based SEDS. However, since the bent structure of OA reduces hydrophobicity, it is more likely to spontaneously disperse and form nanoemulsions with lower PDI when miscible with lower HLB surfactants [59,63]. These results indicate that the balance of the oil and surfactant (HLB) affects the structural stability of the emulsion. Furthermore, the hydrocarbon chain length and unsaturation-induced differences in packing and free volume alter the hydrophobic domains of the oil phase, influencing lycopene dissolution, dispersion, and stability, key considerations for screening SEDS oil matrices.

Turbidity reflects the scattering of light by dispersed droplets in emulsions. In many products, appearance, particularly turbidity, is a critical quality attribute [22]. The OA-based SEDS were mostly transparent, while the MCT- and SO-based SEDS appeared turbid or milky (Table 2). Lycopene loading increased the D_50_ of LL-SEDS during self-emulsification. The SH10S1L formulation had the highest turbidity (0.93 ± 0.01), related to the larger SO-based D_50_ and lycopene-induced extinction effects [37].

The ζ-potential reflects the surface charge on dispersed droplets in emulsions. Higher absolute ζ-potential values indicate stronger electrostatic repulsion between droplets, which prevents aggregation, flocculation, and coalescence, thus maintaining stability [64]. The ζ-potentials were uniformly negative, ranging from approximately −22.90 to −53.70 mV, indicating negatively charged droplet surfaces enabling certain dispersion stability via electrostatic repulsion (Table 2). The MH6S1 and OH6S1 formulations exhibited higher negative ζ-potentials (<−50 mV), indicating stronger interfacial charge stability and reduced aggregation. Conversely, for MCT- or SO-based SEDS (including LL-SEDS) with an HLB of 10.44 and a SOR of 1.00, such as the SH10S1 or MH10S1L formulations, ζ-potentials were relatively low (−22.90 to −32.77 mV), indicating insufficient stability; reduced Brownian motion in spontaneously formed emulsions led to aggregation, increasing SEDS turbidity (Figure 3) and milky emulsion traits (Table 2). Additionally, OA-based SEDS had higher ζ-potentials due to OA’s FFAs. These results demonstrate that SOR-HLB combinations not only affect droplet size and transparency but also alter the surface charge distribution, thereby influencing electrostatic stability [18,26,33,46]. In summary, the type of oil, surfactant properties, and ratios have a decisive influence on the D_50_, dispersibility, turbidity, and stability of LL-SEDS.

### 3.5. Antioxidant Capacity and Lipolysis Dynamics of LL-SEDS

Antioxidant capacity refers to the ability to prevent or mitigate the oxidation of components, particularly oxidizable lipids such as unsaturated fatty acids. High antioxidant capacity is crucial for products whose oxidative degradation may cause rancidity, off-flavors, nutrient loss, or the potential formation of toxic compounds. Among the MCT-based LL-SEDS, the ABTS scavenging rate was higher for the MH10S1L formulation (20.09%) than for the MH6S1L formulation (2.94%). Their ferric ion-reducing capacity differed from their ABTS radical scavenging capacity, suggesting that high-HLB formulations enhance radical capture in the aqueous/solvent phases but have limited impact on metal ion reduction (Table 3) [27,28]. Compared to the MCT-based SEDS, the SO-based SEDS exhibited higher ABTS radical scavenging activity in identical formulations (*p* < 0.05). The SH10S1L formulation demonstrated the best ferric ion-reducing capacity (8.87 mg TE/g DW), indicating good lycopene compatibility and emulsion droplet stability in both the aqueous and solvent phases, favoring lycopene release and antioxidant activity in PBS. The OA-based LL-SEDS displayed no antioxidant activity across formulations, likely due to insufficient lycopene dissolution or droplet structures limiting antioxidant release. Overall, the SH10S1L formulation demonstrated optimal ABTS radical capture and ferric ion reduction (*p* < 0.05).

**Figure 6:**
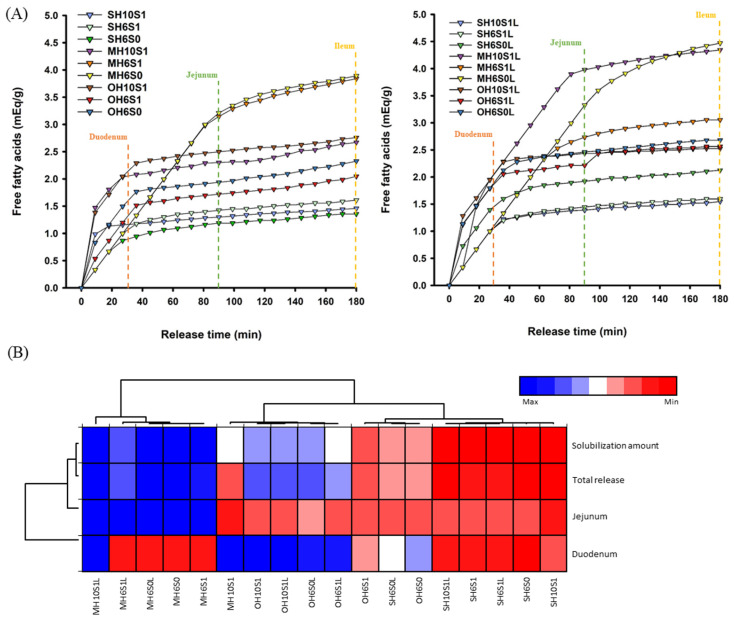
The release of free fatty acids in different lycopene-loaded self-emulsifying delivery systems in simulated digestion. (**A**) Dynamic digestion curve (**B**) Heatmap of solubilization amount in different intestinal segments. M, S, O, H6, H10, S0, S1, and L denote medium-chain triglyceride, sunflower oil, oleic acid, HLB = 6.44, HLB = 10.72, SOR = 0.50, SOR = 1.00, and lycopene-loading, respectively.

In pharmaceuticals, nutraceuticals, and functional foods, SEDS are designed for lipophilic compounds, such as lycopene [8,12,30,38]. Measuring release kinetics in PBS is crucial for assessing the absorption of lycopene in humans [5,26,31,46]. The successful release of lycopene from emulsions and subsequent incorporation into mixed micelles is essential for intestinal epithelial absorption [8,31,65]. Distinct lipolysis behaviors were observed among SEDS; the MH10S1 and SH10S1 formulations, with higher SOR and HLB, dispersed rapidly into small droplets after 30 min in the intestinal segment (duodenum), providing larger lipase-accessible areas and thus exhibiting the fastest lipolysis rates within the first 30 min (Figure 6A) [13,18,26,31,33,46]. However, as droplet surfaces became covered by lipolysis products (e.g., monoglycerides and FFAs) and surfactants, forming dense interfacial layers, subsequent oil diffusion rates slowed, causing the curve to flatten between 30 and 90 min in the jejunum [31,46]. Conversely, the SH6S0 and MH6S0 formulations, with lower SOR, exhibited insufficient initial droplet dispersion, preventing rapid lipolysis. The long-chain fatty acids in the SH6S0 formulation readily formed tight surface layers, causing the curve to flatten at 30 min, while the low viscosity and HLB of the MH6S0 formulation enabled sustained, stable enzymatic hydrolysis, maintaining near-linear rates from 0 to 90 min [30,31,58]. Entering the ileum (90–180 min), accumulated lipolysis products interact with bile salts, gradually covering surfaces, reducing rates, and flattening curves, achieving the highest cumulative release [31,46]. The OA-based SEDS, primarily FFAs, exhibited no lipolysis in simulated digestion; early-segment release depended on the D_50_, with smaller droplets forming high-solubility mixed micelles with bile salts, reducing FFA neutralizability [31,46].

The LL-SEDS exhibited digestion behaviors that differed from the SEDS (Figure 6A). The MH10S1L formulation released rapidly in the duodenal phase (<30 min), which was sustained into the jejunal phase (30–90 min), with the highest cumulative release. The MH6S0L formulation mirrored the steadily increasing trend of MH6S0, with linear rates from 0 to 90 min and the second-highest release. The OH10S1L formulation exhibited a rapid initial release, but remained dynamic after 30 min, possibly due to OA dispersing and neutralizing in intestinal fluids. Lycopene-bile salt complexes may have limited the sustained release (Figure 5). Duodenal and jejunal FFA releases from different LL-SEDS were summed as bioaccessibility estimates (Figure 6B), as lycopene release in the late duodenum and early jejunum favors transport due to optimal bile salt/phospholipid activity and mixing, enhancing mixed micelle formation and enterocyte absorption [66,67,68]. The MCT- and OA-based LL-SEDS were released mainly in the jejunum and duodenum, respectively, while the SO-based SEDS exhibited low cumulative release across segments.

In Figure 6B, the heatmap indicated an increase in the jejunal cumulative release of the MH10S1 formulation upon lycopene loading (MH10S1L). The solubilization amount (duodenal and jejunal) of the MH10S1L formulation reached 3.978 mEq/g, higher than that of MH10S1 (2.300 mEq/g). Furthermore, the MCT-based SEDS performed the best in terms of total release (0–180 min) and solubilization (0–90 min), followed by the OA-based SEDS, with the SO-based SEDS performing the worst. This superiority is attributed to high SOR/HLB MCT-based SEDS generating smaller droplets in intestinal segments, thereby enlarging lipolysis areas and accelerating the reaction of triacylglycerol + 2 H_2_O → 2-monoacylglycerol + 2 FFAs. These products synergize with bile salts/phospholipids to assemble mixed micelles, enhancing lycopene micellar solubilization and transport efficiency [69,70,71,72,73,74].

These results emphasize that SEDS development cannot rely solely on target (lycopene) loading or antioxidant activity; simulated digestion dynamics are essential for inferring bioaccessibility. Our preliminary evaluation of differences in edible oils as SEDS matrices via *in vitro* simulation has confirmed that W/O/W emulsion structures may influence lipolysis efficiency by varying the D_50_, targeting intended intestinal segments. Future research should explore release kinetics, establishing release models for hydrophobic phytochemical delivery as references for oil-based SEDS in functional beverages.

### 3.6. Multivariate Analysis of LL-SEDS 

**Figure 7:**
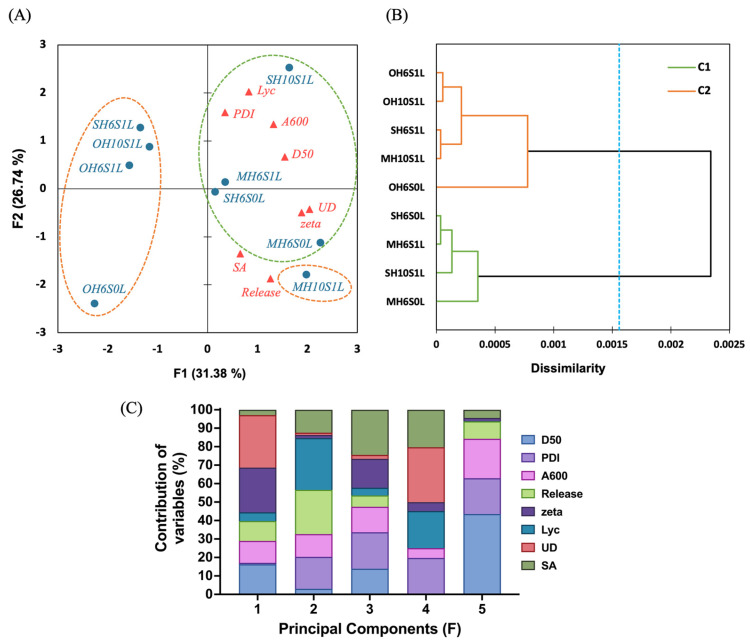
Principal component analysis biplot (**A**), clustering (**B**), and variable contribution (**C**) of emulsion quality and intestinal-segment release on different lycopene-loaded self-emulsifying delivery systems. M, S, O, H6, H10, S0, S1, L, Lyc, PDI, A_600_, D_50_, UD, zeta, SA, and Release denote the medium-chain triglyceride, sunflower oil, oleic acid, HLB = 6.44, HLB = 10.72, SOR = 0.50, SOR = 1.00, lycopene-loading, lycopene loading contents, polydispersity index, turbidity, median droplet size, unsaturation degree, ζ-potential, solubilization amount, and total release amount, respectively.

In the PCA (Figure 7A), principal components F1 and F2 explained 31.38% and 26.74% of the total variance, respectively, together accounting for 58.15%. Clustering distinguished all LL-SEDS into two distinct groups: Cluster 1 and Cluster 2 (Figure 7B). Analyzing LL-SEDS with quality indices, these indices clustered on the right, suggesting the UD of SEDS influences lycopene loading contents (Lyc), affecting W/O/W emulsion ζ-potential (zeta) to lower values for greater stability. Concurrently, the D_50_ and PDI vary by SEDS formulation (e.g., higher in MH6S1L and SH10S1L), depending on the hydrophobicity of the edible oil and HLB of the mixed surfactant (Table 2). Despite lower Lyc, the MH10S1L formulation maintained certain ζ-potential stability; *in vitro* simulated digestion dynamics revealed optimal bioaccessible SA and total cumulative release (Release). This behavior can be explained by MCT-based LL-SEDS having a smaller molecular weight and lower viscosity, which enables them to form nano-grade droplet sizes with higher stability, ranging from −32.67 to −44.17 mV, leading to uniform dispersion, further enhancing lipolysis efficiency and mixed micelle formation afterward, to improve the absorption in the proximal intestine of lycopene [69,70,71,72,73,74]. Thus, the clustering of LL-SEDS on principal component F1 is mainly driven by UD (28.41%), ζ-potential (24.27%), and D_50_ (16.27%), and their clustering on principal component F2 is driven mainly by Lyc (28.06%), Release (23.94%), and PDI (17.27%, Figure 7C). Additionally, despite their smaller D_50_ (181.70–322.03 nm) and lower PDI (0.29–0.80), OA-based LL-SEDS exhibited low lycopene content and cumulative release, suggesting that SEDS and LL-SEDS cannot form smaller emulsion sizes alone but require practical evaluation in simulated release environments.

## 4. Conclusions

The HLB and SOR significantly influence lycopene solubility, emulsion characteristics, and lipolysis behavior, with MCT-based LL-SEDS achieving superior bioaccessibility during simulated intestinal digestion (3.978 mEq/g). These systems leverage the synergistic effects of nonionic surfactants to form stable W/O/W emulsions (D_50_: 280.73–572.27 nm, ζ-potential: −32.67 to −44.17 mV, PDI: 0.40–0.56) through low-energy self-emulsification, providing a sustainable alternative to high-energy emulsification methods for food-grade carriers. SO-based SDS exhibited the highest antioxidant activities (ABTS radical scavenging: 13.18–21.80%, FRAP: 2.94–8.87 mg TE/g DW), highlighting their potential for functional foods. Multivariate analyses revealed the critical interplay between oil type, surfactant properties, and release dynamics, emphasizing the need for balanced hydrophobicity to optimize delivery. Among them, the MCT-based LL-SEDS composed of MCT, T80, and S80 at a 5:3:2 ratio exhibited the optimal comprehensive characterization. These findings establish a framework for developing low-energy LL-SEDS for hydrophobic bioactives, with applications in fortifying beverages, sauces, and other functional food matrices. Future research should focus on exploring *in vivo* bioavailability, sensory attributes, and scalability to ensure alignment with food industry requirements, including shelf-life stability and consumer acceptability.

## Figures and Tables

**Table 1:** Formulation and lycopene content of lycopene-loaded self-emulsifying delivery system.

Formulation	SO	MCT	OA	T80	S80	Theoretical Loading of Lycopene	Real Loading of Lycopene	Bias
Unit	%	%	%	%	%	mg/g	mg/g	%
MH6S0	–	66.67	–	6.67	26.67	–	–	–
MH6S1	–	50.00	–	10.00	40.00	–	–	–
MH10S1	–	50.00	–	30.00	20.00	–	–	–
MH6S0L	–	66.67	–	6.67	26.67	11.99 ± 0.03 ^i^	23.99 ± 0.73 ^c^	100.08
MH6S1L	–	50.00	–	10.00	40.00	16.78 ± 0.08 ^e^	28.99 ± 0.47 ^a^	72.77
MH10S1L	–	50.00	–	30.00	20.00	15.26 ± 0.02 ^g^	22.32 ± 0.16 ^d^	46.26
SH6S0	66.67	–	–	6.67	26.67	–	–	–
SH6S1	50.00	–	–	10.00	40.00	–	–	–
SH10S1	50.00	–	–	30.00	20.00	–	–	–
SH6S0L	66.67	–	–	6.67	26.67	17.75 ± 0.10 ^d^	21.85 ± 0.15 ^d^	23.10
SH6S1L	50.00	–	–	10.00	40.00	21.10 ± 0.14 ^a^	27.15 ± 0.94 ^b^	28.67
SH10S1L	50.00	–	–	30.00	20.00	19.58 ± 0.08 ^b^	29.87 ± 0.16 ^a^	52.55
OH6S0	–	–	66.67	6.67	26.67	–	–	–
OH6S1	–	–	50.00	10.00	40.00	–	–	–
OH10S1	–	–	50.00	30.00	20.00	–	–	–
OH6S0L	–	–	66.67	6.67	26.67	13.57 ± 0.04 ^h^	14.53 ± 0.81 ^e^	7.07
OH6S1L	–	–	50.00	10.00	40.00	17.96 ± 0.09 ^c^	27.23 ± 1.05 ^b^	51.61
OH10S1L	–	–	50.00	30.00	20.00	16.45 ± 0.03 ^f^	22.32 ± 0.22 ^d^	35.68

Data are presented as means ± standard deviations (*n* = 3). Different letters (a–i) indicate statistically significant differences in the same column (*p* < 0.05). M, S, O, H6, H10, S0, S1, L, MCT, OA, SO, T80, and S80 denote medium-chain triglyceride, sunflower oil, oleic acid, HLB = 6.44, HLB = 10.72, SOR = 0.50, SOR = 1.00, lycopene-loading, medium-chain triglycerides, oleic acid, sunflower oil, Tween 80, and Span 80, respectively.

**Table 2:** Quality assessment of different lycopene-loaded self-emulsifying delivery system.

Formulation	D_50_ (nm)	PDI	A_600_	Appearance	ζ-Potential (mV)
MH6S0	380.17 ± 19.13 ^e^	0.51 ± 0.08 ^def^	0.02 ± 0.00 ^hi^	transparent	−34.70 ± 2.44 ^bc^
MH6S1	488.30 ± 48.33 ^b^	0.86 ± 0.19 ^a^	0.01 ± 0.01 ^i^	transparent	−53.70 ± 3.81 ^i^
MH10S1	260.37 ± 4.02 ^h^	0.33 ± 0.04 ^gh^	0.31 ± 0.00 ^c^	milky	−22.90 ± 0.26 ^a^
MH6S0L	572.27 ± 11.73 ^a^	0.54 ± 0.07 ^de^	0.12 ± 0.00 ^f^	turbid	−37.07 ± 0.93 ^c^
MH6S1L	446.83 ± 17.50 ^c^	0.56 ± 0.03 ^cd^	0.17 ± 0.00 ^e^	turbid	−44.17 ± 1.68 ^ef^
MH10S1L	280.73 ± 2.25 ^h^	0.40 ± 0.04 ^efgh^	0.46 ± 0.01 ^b^	milky	−32.67 ± 1.70 ^b^
SH6S0	428.10 ± 14.54 ^cd^	0.48 ± 0.13 ^defg^	0.01 ± 0.01 ^ij^	transparent	−42.97 ± 2.44 ^def^
SH6S1	446.60 ± 9.60 ^c^	0.60 ± 0.01 ^cd^	0.03 ± 0.01 ^gh^	transparent	−45.60 ± 3.16 ^fg^
SH10S1	491.87 ± 10.64 ^b^	0.45 ± 0.06 ^defg^	0.31 ± 0.00 ^c^	milky	−32.27 ± 0.31 ^b^
SH6S0L	414.30 ± 9.92 ^d^	0.48 ± 0.06 ^defg^	0.10 ± 0.02 ^f^	turbid	−40.53 ± 1.45 ^d^
SH6S1L	350.93 ± 5.84 ^f^	0.47 ± 0.08 ^defg^	0.17 ± 0.00 ^e^	turbid	−44.77 ± 1.60 ^fg^
SH10S1L	414.30 ± 3.40 ^d^	0.52 ± 0.03 ^def^	0.93 ± 0.01 ^a^	milky	−32.77 ± 1.06 ^b^
OH6S0	215.70 ± 3.15 ^i^	0.33 ± 0.02 ^gh^	0.00 ± 0.00 ^j^	transparent	−40.37 ± 1.15 ^d^
OH6S1	181.70 ± 1.71 ^j^	0.38 ± 0.01 ^fgh^	0.01 ± 0.01 ^ij^	transparent	−52.93 ± 0.64 ^i^
OH10S1	287.37 ± 20.77 ^h^	0.80 ± 0.15 ^ab^	0.02 ± 0.00 ^hi^	transparent	−43.40 ± 0.62 ^def^
OH6S0L	212.17 ± 1.92 ^i^	0.29 ± 0.03 ^h^	0.04 ± 0.00 ^gh^	transparent	−41.07 ± 1.59 ^de^
OH6S1L	322.03 ± 5.38 ^g^	0.53 ± 0.01 ^def^	0.04 ± 0.00 ^g^	transparent	−47.63 ± 2.72 ^gh^
OH10S1L	315.93 ± 18.66 ^g^	0.69 ± 0.04 ^bc^	0.26 ± 0.02 ^d^	turbid	−49.17 ± 1.55 ^h^

Data are presented as means ± standard deviations (*n* = 3). Different letters (a–j) indicate statistically significant differences in the same column (*p* < 0.05). M, S, O, H6, H10, S0, S1, L, D_50_, PDI, and A_600_ denote medium-chain triglyceride, sunflower oil, oleic acid, HLB = 6.44, HLB = 10.72, SOR = 0.50, SOR = 1.00, lycopene-loading, median droplet size, polydispersity index, and turbidity, respectively. The appearance is followed A_600_ value with <0.05, 0.05–0.1, 0.1–0.3, and >0.3 denote transparent, bluish/semi-transparent, turbid, and milky, respectively.

**Table 3:** Antioxidant activity of different lycopene-loaded self-emulsifying delivery system.

	ABTS (%)	FRAP (mg TE/g DW)
MH6S0L	ND	2.70 ± 0.03 ^d^
MH6S1L	2.94 ± 0.77 ^c^	2.63 ± 0.03 ^d^
MH10S1L	20.09 ± 1.24 ^a^	0.67 ± 0.01 ^f^
SH6S0L	13.18 ± 1.10 ^b^	5.60 ± 0.04 ^b^
SH6S1L	19.85 ± 1.61 ^a^	2.94 ± 0.02 ^d^
SH10S1L	21.80 ± 1.04 ^a^	8.87 ± 0.07 ^a^
OH6S0L	ND	3.65 ± 0.09 ^c^
OH6S1L	ND	3.79 ± 0.13 ^c^
OH10S1L	ND	0.88 ± 0.05 ^e^

Data are presented as means ± standard deviations (*n* = 3). Different letters (a–f) indicate statistically significant differences in the same column (*p* < 0.05). M, S, O, H6, H10, S0, S1, L, and ND denote medium-chain triglyceride, sunflower oil, oleic acid, HLB = 6.44, HLB = 10.72, SOR = 0.50, SOR = 1.00, lycopene-loading, and not detected, respectively.

## Data Availability

The original contributions presented in the study are included in the article, further inquiries can be directed to the corresponding author.
